# Understanding Appearance-Enhancing Drug Use in Sport Using an Enactive Approach to Body Image

**DOI:** 10.3389/fpsyg.2017.02088

**Published:** 2017-11-29

**Authors:** Denis Hauw, Jean Bilard

**Affiliations:** ^1^Institute of Sport Sciences, University of Lausanne, Lausanne, Switzerland; ^2^Faculty of Sport Science of the University of Montpellier, Montpellier, France

**Keywords:** enaction, embodiement, body awareness, situatedness, participatory sense-making, developmental explanatory thesis, substance use, adolescent anxiety

## Abstract

From an enactive approach to human activity, we suggest that the use of appearance-enhancing drugs is better explained by the sense-making related to body image rather than the cognitive evaluation of social norms about appearance and consequent psychopathology-oriented approach. After reviewing the main psychological disorders thought to link body image issues to the use of appearance-enhancing substances, we sketch a flexible, dynamic and embedded account of body image defined as the individual’s propensity to act and experience in specific situations. We show how this enacted body image is a complex process of sense-making that people engage in when they are trying to adapt to specific situations. These adaptations of the enacted body image require effort, perseverance and time, and therefore any substance that accelerates this process appears to be an easy and attractive solution. In this enactive account of body image, we underline that the link between the enacted body image and substance use is also anchored in the history of the body’s previous interactions with the world. This emerges during periods of upheaval and hardship, especially in a context where athletes experience weak participatory sense-making in a sport community. We conclude by suggesting prevention and intervention designs that would promote a safe instrumental use of the body in sports and psychological helping procedures for athletes experiencing difficulties with substances use and body image.

## Introduction

It is easy to understand the importance of presenting a good image: a good self-presentation enhances employability and recognition by others, tipping the balance toward positive evaluation (e.g., Nicholls, 1984; Haasler, 2013). Body image, defined as the visual appearance we present to others, is a tool for the non-verbal aspect of self-presentation (e.g., Knapp et al., 2014), and it is generally what attracts attention in social interactions (e.g., Featherstone et al., 1991; Richmond et al., 2008). Moreover, the nearly universal need to be included in society is thought to be one of the main drivers of the preoccupation with body image or appearance (e.g., Kaufmann, 2005; Grogan, 2008).

Participation in sports or physical activities is an obvious way to reach this goal. Regular sports participation shapes the body and helps the individual to display agility, availability, or youthfulness. But it also requires effort and the physical outcomes are not always immediate and durable. Artificial substances are potential accelerators in developing a fit and attractive body and then maintaining the effects. Thus, they can be considered as a means for bridging the gap between the desire to shape the body image and the constraints to doing so (e.g., Bodin et al., 2005).

However, we suggest that this psychosocial facet of body image has not been fully explored (e.g., Hauw and Bilard, 2012; Bilard and Hauw, 2016): we present four arguments to support this contention that we will develop in the following text. First, body image is not only body presentation to others but also what a person experiences through the sensory system, as suggested by the detailed observations of Schilder et al. (1968). Second, body image is a poly-semantic concept that is too often used while ignoring the dimensions of the personal and historical construction in personal development (e.g., Wallon, 1949/1983; Dolto, 1984). Third, body image is not an inert and cognitive psychological disposition that results from cognitive, emotional or social evaluation approached in a binary manner, such as dissatisfied or not, pathological or not, and dysmorphobic or not. Instead, it covers multiple flexible, dynamic and embedded propensities to act and experience in situations that are meaningful for an individual even though they are mainly transparent to the cognitive self. Body image in this sense refers to the large experiences of the body in situation, including the means for achieving access to the world (e.g., Noé, 2012) and the feeling of truly being there (e.g., Clark, 1997). Fourth, body image does not become impaired randomly in an individual’s development. Vulnerabilities in this aspect of the self emerge when specific events in life periods seem to transform the sense-making of a person’s body image (e.g., Nasio, 2007; Hauw, 2017). This last point suggests that societal pressure for appearance or ideal standards are not continuous but arise in specific situations where body image as a part of an individual’s complex sense-making system is involved in adaptation processes.

Thus, in this article we sketch an enactive account of body image showing that the use of appearance-enhancing drugs is better explained by the sense-making related to the experienced body that people bring forth in given situations (including social demands) rather than solely by the evaluation of social demands about appearance or the consequent pathological body image in reference to these norms. Sense-making is the core contribution of the enactive approach to the modern cognitive sciences. It considers human activity as embedded, embodied, extended, and enacted (Rowlands, 2010; Hutto and Myin, 2013). As we will develop later in this text, this approach emphasizes that individuals, instead of evaluating information from an external world, create meaningful worlds of acting, feelings, and thoughts when involved in specific situations (e.g., von Uexküll et al., 1984; Froese and Di Paolo, 2011; Froese and Stewart, 2012). From an enactive approach, this involvement is fundamentally an embodied interaction with the environment. The consequence is that body image is always an accomplishment in situation. We thus use an enactive account of body image to build new bridges to understanding the use of appearance-enhancing substances and describe, illustrate and define the different properties of body image that elite or amateur athletes bring forth in situation.

In the following sections, we present our main theoretical assumption, which is twofold: first, we claim that the use of appearance-enhancing drugs is linked to the general sense-making that individual athletes bring forth in relation to performance and appearance requirements in order to be recognized as belonging to a sports community (participatory sense-making). Second, we claim that this use and its links to sense-making are anchored in specific life periods where body experience was weakened and thus results from the history of the body’s previous interactions with the world. Hence, we show how this relationship to appearance-enhancing substances may be differently embodied as it shapes personal identity, including body image as part of the situated self.

In the first section, we review how body image and the use of appearance- and performance-enhancing substances are considered in the light of current psychological and cognitivist approaches. We then take an enactive approach to human activity and examine how the enactive body image is involved in sports practice and why and which appearance-enhancing drugs are likely to be used. We then focus on specific periods of development when body image is weakened. We conclude by reflecting on prevention measures that would promote the safe instrumental use of the body and on the use of psychological services to help athletes experiencing difficulties with substance use linked to body image.

## Psychological Approach to The Relationship Between Body Image and Substance Use

Body image as a concern about body appearance currently affects people of all ages. For example, it was recently shown that between 20 and 70% of children under the age of six experience body dissatisfaction (Tatangelo et al., 2016). An estimated two thirds of adolescents may be dissatisfied with their bodies, equally divided between weight loss and muscle gain concerns (McCabe and Ricciardelli, 2004). With aging, the body inevitably moves women and men away from youthful thinness and ideal muscularity, but research has shown that body dissatisfaction remains relatively stable across the life span (Grogan, 2008). Tiggemann (2004) suggested that the importance of body appearance for adults decreases with age. He then went on to show that, although the body deteriorates with age, people remain equally dissatisfied, and he concluded that over time they tend to engage in control strategies to reduce the importance to them. McCabe and Ricciardelli (2004) showed that adult men evidence a strong desire to lose weight as they get older.

Other research, mostly focused on adolescents, has demonstrated that the use of substances like nutritional supplements or anabolic steroids is linked to body dissatisfaction (e.g., Yager and O’Dea, 2014). A survey conducted with more than 14,000 adolescents in the United States showed that the use of performance-enhancing substances (methamphetamines, steroid pills/injections, and diet pills/powders/liquids) was linked to feelings of sadness or hopelessness and perceptions of overweight (Thorlton et al., 2012). Phillips et al. (2006) examined the clinical features of body dysmorphic disorder in adolescents and adults and found that adolescents were preoccupied with many aspects of their appearance, most often skin, hair, and stomach. Among these adolescents, 94.3% reported moderate, severe, or extreme distress. These body dysmorphic adolescents also experienced high rates and levels of impairment in school, work, and other aspects of psychosocial functioning. Hildebrandt et al. (2011b), who validated the appearance and performance enhancement drug use schedule, showed that the scales correlated with DSM-IV diagnoses, aggressiveness, impulsivity, and eating disorders. Hildebrandt et al. (2011a) identified three phenomenological features associated with increased heath risks and pathology: (a) polypharmacy to change outward appearance or increase personal achievement, (b) body image disturbance, and (c) rigid practices for diet and exercise. Other research showed that muscle dysmorphia was linked to a large pharmacopeia (e.g., Murray et al., 2012). Body checking among males (Walker et al., 2009) was correlated with weight and shape concerns and predicted the use of appearance- and performance-enhancing drugs. Foster et al. (2015) recently suggested that muscle dysmorphia, described as a misconstrued body image in which individuals who interpret their body size as both small and weak despite looking normal or highly muscular, might be considered as an addiction to body image (ABI), using Griffiths’ (2005) model. To summarize, the psychological approach to body image offers a description of body dissatisfactions as pathological, linked to mental concerns or social evaluations regarding weight and shape and leading to substance use, dieting, and rigid or compulsive exercise.

## Reconsidering Body Image With an Enactive Approach

The enactive approach suggests that human activity is based on embodiment and mind processes that progressively shape the way people interact with their environment (e.g., Rowlands, 2010). One of the most important assumptions is the following: by moving around and with the world, people make sense of this world (sense-making) (e.g., Stewart et al., 2010; De Jaegher, 2013). This sense is “written” in the body and in return determines how they will act and be modulated by events (Varela et al., 1991). Thus, body image in the enactive approach could be understood as emerging in close relation to the type of practice, with the many types of practice each creating a field of possibilities as individuals interact with incoming situations and ground their activities (Sheets-Johnstone, 1999). Thus, instead of considering the discrepancy between ideal and actual body image, as suggested by the cognitivist approach (e.g., Marsh, 1999; Leit et al., 2002), this approach places greater emphasis on the activities that have shaped body image and the possible interactions with the diversity of lived situations that it brings forth. In this article, we will not explain all the pillars that support the enactive approach or those that have been used for the analysis of sports activity, including doping (for example see Hauw and McNamee, 2015; Hauw and Mohamed, 2015; Hauw, in review). But we will underscore two important assumptions that are included in all the variations of enactivism (Hutto and Myin, 2013): autopoiesis and the developmental-explanatory thesis.

The autopoietic thesis suggests that “mentality is something that emerges from the self-organizing and self-creating activities of living organisms” (Hutto and Myin, 2013, p. 32). Mentality as a synonym for “the self in situation” includes body image as an embodied part of a situated approach to personality (e.g., Doris, 2002; Gibbs, 2006). Body image is thus grounded in self-organizing and creating activities that relate to meaning or sense-making and norms that organisms enact or bring forth on the basis of their autonomy (e.g., Thompson, 2010; McGann et al., 2013; Colombetti, 2014; McGann, 2014). Autonomy thus differs from the idea that social ideal or norms such as pressure for appearance explain individual behavior because each person builds his or her own world of feelings, thinking, and acting.

The developmental-explanatory thesis argues that the “mentality-constitutive interactions are grounded in, shaped by, and explained by the history of an organism’s previous interactions” (Hutto and Myin, 2013, p. 8). Hence, “a prolonged history of interactive encounters is the basis of creatures’ current embodied tendencies, know-how and skills” (Hutto and Myin, 2013, p. 9). This second thesis suggests that body image as a propensity to act is made up in part by the legacy of past interactions whose outcomes may emerge in future situations, as well as in current or new adaptations to a situation.

Embodied tendencies and their autonomous properties and historical relatedness thus can be used to understand the concept of body image in an enactive way. Let us recall Schilder’s suggestion that body image results from the interaction of three layers (Schilder et al., 1968): (a) a biological layer well-known through the concept of body schema (e.g., Gallagher, 1986), (b) a libidinal layer that gives emotional coloring to the experiences associated with body perception (e.g., Nasio, 2007), and (c) a social layer commonly delimited by social appearance (Schilder et al., 1968). This author also argued that body image is not a lifeless representation of self, but is instead composed of the various dynamic phenomenological gestalts that describe the adaptive nature of the relationships between humans and their environment. Compatible with an enactive perspective, body image in Schilder’s conception captures the sense-making processes in embedded and embodied human activity (Schilder et al., 1968). This body image thus moves in relation to life events, as Schilder (Schilder et al., 1968) and Luria (1968, 1987) suggested and, more recently, as the clinical descriptions of injury and reconstruction from Sacks (1987) illustrated. Hence, body image is not only the social image that a person reflects, not merely an internal representation of self. Rather, it corresponds to the various body propensities to act and experience in situation, thus, to the various selves (organic, emotional, cognitive, social) that emerge in interaction with the environment (Sheets-Johnstone, 1999; Smetacek and Mechsner, 2004).

Many theories in psychoanalysis and psychopathology have explored the history of the constitution of body image. One of the key phenomena is the so-called mirror stage (e.g., Wallon, 1949/1983; Lacan, 1966; Dolto, 1984). For Lacan (1966), the image of the body in the mirror, or the “specular image,” evokes jubilation in the infant who sees himself as a human figure and a harmonious whole. This entity contrasts with the feeling of motor immaturity and internal corporeal fragmentation. In the mirror, the child has the impression of being different and separate from others – of “being One – which prefigures the I when she will later on speak in her own name.” This specular image gives the infant the feeling of being a coherent and harmonious unit that prefigures the future Me. Last, the discrepancy between what the child sees in the mirror and what she feels – that is, the discrepancy between the harmony of the reflected image and the agitation of internal sensations that dominate an immature body – helps us to understand our lifelong fascination with body image as appearance. When the child discovers, at about two and a half years old, that this image in the mirror is not really him, that an irreducible gap separates the “unreality” of his specular image from the “reality” of his person, it is a profound “shock.” According to Dolto (1984), in reaction to this disenchantment, the child abandons these unconscious images of the body that arise from proprioceptive, interoceptive and erogenous sensations and begins to overvalue the flattering images of appearance. Henceforth, images of the “viewed body” will take precedence over images of the “lived body” that will be relegated to the unconscious. In later years, the adult will tend to neglect the internal and sensory life of her body and focus on her appearance. More recently, Nasio (2007) suggested that body image is a global synthesis elaborated by our physical sensations and the image of our figures in the mirror (other people always being the mirror of ourselves by identification and their regard). This “dynamic” body image is built, developed and regenerated throughout a lifetime and thus should be understood as situated body availabilities regarding social and individual constraints.

For Schilder, these different facets of body image emerge into awareness in specific situations (Schilder et al., 1968). For example, when a person participates in a physical activity or sport, body image is involved because the activity of the body perturbs the image along physiological, psychological, or sociological dimensions. This perturbation maintains or transforms the body image with activity. An example is the athlete who feels stronger than usual after a fitness session. The activity in the fitness session has supported body image as a propensity to be strong in the situation. In a contrasting example, a sedentary person may feel that is too difficult to exercise after experiencing muscle soreness. The body image and propensity to act are impaired by muscle sensations. In addition, psychoanalysts contend that an unconscious level in the organization of body image may emerge when the perturbation is strong. This level corresponds to narcissistic impulses regarding the legacy of repression in infancy (Freud, 1914/2012). It could also be understood as a process of adaptation related to the developmental history, particularly the relationship between mother and child (e.g., Bowlby, 1951; Anzieu, 1974; Winnicott, 1975). Hence, body image may also express a unidirectional propensity to act without conscious control. For example, some athletes may have the feeling of not being fit after a short period of recovery. They feel an irresistible physical need to train intensively again and again.

This enactive approach to body image using interdisciplinary evidence offers deeper insight into the psychological processes involved in appearance-enhancing drug use in the context of sport because the practices themselves are typical situations in which body image is particularly likely to be disturbed. As Schilder suggested, it would be interesting to study the movements that modify body image and those that do not (Schilder et al., 1968).

## Enactive Body Image: Awareness and Appearance

Most of time, body image is an efficient propensity to act. People have learned and incorporated how their bodies should be attuned to the motor, energetic and social requirements of their activity (e.g., Goffman, 1998; Novacheck, 1998; Davids et al., 2008). However, there are also several situations in which body image emerges into awareness. Although a muscular body has become the symbol of willpower, energy, and control for men (e.g., Duret, 1999; Grogan, 2008) and thinness is now the ideal body shape for women (e.g., Vigarello, 1975; Martin and Gentry, 1997), various other ways of enacted body image can be identified and might be tested empirically. These include such specific situations as fatigue, sexual excitement, pain/pleasure, sickness, mental illness, stress, and physical challenges (e.g., Schilder et al., 1968). Pollio et al. (2006) identified the “whens” and “whats” of such awareness with adults in eight typical daily body experiences (i.e., using, sensing and presenting the body; pregnancy and sexuality; changes over time; awareness of the meaning of some events; awareness of others and of affect). They also identified three modes of experiencing the body: engagement (vitality and activity), corporeality (instrument and object), and interpersonal meaning (appearance and expression of self). These types of body awareness emerge in relation to the requirements of the situation and suggest in which cases a modification in body image can regulate human activity and thus be enacted in a specific way.

Some of these cases appear to be particularly linked to sports or physical activity situations. We can also argue that there is a circular relationship between sports and body image: by engaging in sports, people enact a specific body image and transform it as well as their way of practicing their sport (e.g., Rossi and Zoccolotti, 1979). Schilder observed that human beings are confined and disturbed by body image (Schilder et al., 1968). One of the motivations that drive them to transform it is the desire to overcome its rigidity. According to Schilder, an effective way to dissolve and soften the rigidity of the postural model of the body is dance (Schilder et al., 1968): the old framework remains, but a new frame is built upon it. Movement is elaborated from a relatively rigid primary figure, which then seems to be partly loosened and dissolved; the body is then inserted into one of the primary attitudes adapted to the physical or musical environment. Dance movement often uses postural reflexes that are not fully conscious. Dance can be the means of activating what is most ancient in us, our most original corporeal sensations (i.e., the pictogrammatic background, Aulagnier, 1975). The dancer in the ecstatic experience of “my body in the world” has escaped the limits of Me and I, and others no longer exist. But this is social depersonalization to the benefit of life itself: what is within us is enlarged to the empathic perception of the universe.

In sports and physical activities, the best known examples of the issue of body image are bodybuilding and fitness, as they display the two modes identified by Pollio et al. (2006): appearance and vitality. In bodybuilding, the propensity is to display a particular body shape. Appearance is one of the main outcomes with the focus drawn to the shape of the muscles. However, recent research has also shown that bodybuilders strive for high performance in terms of exercises, repetitions of series, and weight lifted (Coquet, 2016). Here, the use of polypharmacy to reach these goals is well-known (e.g., Cooper et al., 1996; Augé and Augé, 1999; Sagoe et al., 2014). The fitness practices that have recently emerged make sense of “body vitality,” expressed by the capacity to make repeated intensive efforts, drawing on the aerobic metabolism (e.g., cardio kickboxing, aeroropes) and pushing physical limits (e.g., Crossfit, Mud Day). These kinds of fitness sports are midway between bodybuilding and conventional sports, because the twofold propensity of the body (performance and appearance) are more salient. In terms of Pollio et al.’s (2006) identified modes of body awareness, fitness sports require an awareness of the body as an efficient instrument that should also have a certain appearance. In these cases, the use of appearance-enhancing drugs is linked to the possibility of meeting performance requirements, of making the body efficient in relation to the values promoted by the practice (e.g., via stimulants or pain-killers). And when practitioners meet this requirement, they have “the appearance” of belonging to a community such as “ultratrail finisher.” The appearance is here linked to the status of performer.

In established sports, there are “thin-type” sports such as rhythmic gymnastics, ski jumping, long-distance running and ballet: the two requirements of body propensity are to display perfect body aesthetics and to be light or fit. Rugby, American football, and rowing require the same propensity, although opposite in terms of body shape. There are also other intermediary sports for which a typical body shape is needed and has to be emphasized in relation to performance: tall and fit for surfing and combined events in track and field, wide shoulders but overall slimness for swimming, wide shoulders and exaggerated development of arm and shoulder muscles in artistic gymnastics. Here, the drugs used for appearance are also linked to the performance requirements, such as anabolic steroids or growth hormone for body growth or diuretics for weight loss.

Hence, each sport emphasizes more or less phenomenal propensities of the body to act in certain directions, intertwined with the figures of body presentation. Thus, appearance is linked with a certain mode of body-efficient activity that underlines what a person is able to do and who she is. The different layers of enacted body image operate within these activities in which appearance is closely connected to performance.

## Adaptations of Body Image and Attraction to Appearance-Enhancing Drugs

As we mentioned, this specific perspective on body image also raises the issue of the process of adaptation to the situation. Adaptation requires effort and perseverance, and it is time consuming and sometimes painful (e.g., Starkes and Ericsson, 2003). It is of particular concern for the individual because it is related to two norms that are currently depreciated: a long time period and high and sustained investment (Kaës, 2015). Thus, three rules for more easily enacting adaptations can be formulated. The adaptations should meet the requirements for (a) performance and (b) appearance, and (c) meeting these requirements should not take too much time. For the physical activities and sports we mentioned in the previous paragraphs, performance has always been in one way or another linked to appearance. Attaining high performance implicates components of the biological layer of body image: postures and balance for bodybuilding, general motor coordination for fitness sports, specific skills or energetic capacity for conventional sports. Thus, these dimensions are necessary conditions for adaptation but are not sufficient: body image adaptations should also meet the cultural values of the activity that make belonging to a sports or other community visible to others.

The shape of the body is generally evident but certain habits or capacities to act with the body in a certain manner are challenging. Once again, the concept of the necessary but not sufficient condition applies – thus: don’t judge a book by its cover. Also, a person should not remain outside of a community of practice for too long, as the risk is isolation or negative attitudes or reactions (stigma). Yet a certain standard of performance and appearance must be attained to become eligible as a participant in a community (e.g., Lave and Wenger, 1991). Enactivists call this “participatory sense-making” and define it as “the coordination of intentional activity in interaction, whereby individual sense-making processes are affected and new domains of social sense-making can be generated that were not available to each individual on her own” (De Jaegher and Di Paolo, 2007, p. 13). This rule protects the person from social rejection but also encourages all means that accelerate entry into the community. It is here that drugs enter the scene to sustain the adaptations of body image and the unreasonable aspects of sports (e.g., overtraining, exercise addiction).

The last rule is associated with the time needed to transform body image. As suggested by deliberate practice theory, considerable training, effort, suffering, and time are required before transformation is observable (e.g., Starkes and Ericsson, 2003). But when the individual feels a pressing need for change, it is very hard to wait for it to become noticeable, for both psychological and social reasons. An easy solution is to take drugs that accelerate the physical transformation with less effort and sustained effects. This has often been observed in young talented athletes who are tall but unable to gain enough body mass despite a customized program. It has also been observed in professional athletes who are incapable of transforming their bodies despite a high volume of mass-building exercise. Coaches become impatient in this situation and the athletes are thus vulnerable to body-enhancing substances as a means to affect not only performance but also their identity in relation to their place in the sport (e.g., Field et al., 2014). Till et al. (2015) observed this vulnerability in adolescent rugby players who turn to testosterone consumption to meet the requirements for joining a professional team. We also can argue here that the appearance-enhancing substances used here are those that not only transform the body (e.g., muscle gains with growth hormones and anabolic steroids, weight loss with diuretics), but also those that offer the possibility of performing with the body (e.g., amphetamines, pain killers). Thus multi-consumption is progressively required in relation to the performance and appearance requirements that need to be enacted within a short time.

In addition to these three rules, the greater the requirement for a phenomenal performance and body appearance, the more crucial it becomes to take body image-enhancing drugs that help to enact this sense-making. Research has clearly demonstrated this in bodybuilding and fitness centers, where the percentage of steroid and stimulant users can be very high as these individuals struggle to render the body capable of acting as required in this situation (not only in the sense of appearance but also for, as an example, the repetition of exercises) (e.g., Simon et al., 2006; Stubbe et al., 2013). Similar results have been observed for sports requiring a very thin body, like long-distance running or cycling, with high use of fat-burning substances that also allow to perform easily with a lightweight body (e.g., Check Hayden, 2008). Hence, the performance-enhancing substance meets the body appearance requirement in order to produce the right body in the right place.

## Adolescent Anxiety and Upheavals in Body Image

Just as body image provides the propensity to act and experience, which can be completely assumed or normalized by a person in all the shades of possibilities, there are also situations where very high anxiety accompanies these propensities. This is particularly the case in adolescence, a time when the body is sometimes seen as the enemy (Birraux and Marty, 2013). The specular images are precarious, and body image is transforming during the pubertal growth spurt and the modifications in identifications (i.e., end of identification with parents, absence of secondary identification or idealistic model) or in sense of social participatory. These upheavals in body feeling are often associated with anxiety (e.g., Crocker et al., 2003; Cascone et al., 2011). The loss of the child’s body and the conquest of the adult’s are too disturbing a route for some adolescents and defense mechanisms emerge. Adolescents and young adults may become lost in the interaction with the situation, and this is apparent when body appearance causes body dysmorphic disorder. In most cases, this stage is transactional: for adolescents, adaptation means finding limits and a secure stance, which is inherent to this period of development. Many of the calls to the French hotline Ecoute Dopage have been examples of this type of case (Bilard et al., 2011; Mohamed et al., 2013; Bilard and Hauw, 2016).

A typical example is the adolescent’s call to explain that he needs intensive strength building exercises in a fitness center in order to modify and correct physical shortcomings. In most cases, these adolescent callers describe a situation of profound unease, social rejection from peers, and drug use associated with the practice. Their calls illustrate the impact of a disturbed body image at a specific moment and the re-enactment of the body image. There are no strong rational reasons for their perturbations, but these adolescents are clearly lost in their interaction with the situation. Yet despite this, they generate activities and are clearly trying to bring forth a new world. Unfortunately, these activities do not transform the situation sufficiently.

As we have mentioned, this kind of lostness is in most cases the result of transformations related to adolescence and thus mostly temporary. However, it can also reveal a perturbation in the propensities of body image profoundly grounded in the history of childhood and later adolescence and then into adulthood. One hypothesis to explain this perturbation is linked to attachment theories. According to Bowlby (1951) and Winnicott (1975), these adolescents were not completely supported in the development of their body image during childhood. They were not scaffolded in their development, with impaired holding, handling or object-presenting (Delourmel, 2002). According to the dynamic model of attachment and adaptation, these adolescents were not appropriately protected and comforted in childhood (i.e., anxious children – types A and C, in contrast to secure children – type B) (Pierrehumbert, 2005; Crittenden, 2008). This made it harder for them to learn to predict, identify and adjust to threats like the transformation of body image and attracted them to messages to use enhancing substances. This is particularly true when the regulation of arousal (i.e., body) and attention (i.e., mind) are not well-developed. When parents do not manage arousal in their babies and children, negative effects are later observed, including the long-term inability to self-regulate arousal (Crittenden, 2008).

Some of the calls to Ecoute Dopage come from parents that feel anxious about their adolescent’s behaviors and substance use. In each case, the situation starts from the same issue of body image. But here, the anxiety and feelings of discomfort have spread to the parents. The depreciation of body image is not only reflected in the adolescents’ dispositions but also in what the parents did with them. There is also the fear of separation, particularly when it is linked to the transformations of the child’s body to that of an adult.

The developmental-explanatory thesis of enactivism appears in a concrete form in adolescents who were anxious children (types A and C) and who must now, in adolescence, adapt by coming to safety and comfort (Crittenden, 2008). It is particularly difficult for them to adapt to this perturbation because they have to build new meanings for their interactions with situations that are stressful and uncomfortable. Thus, for these adolescents a disturbance in body image is potentially more than a temporary adaptation. Another point should also be mentioned within this more complicated scenario: studies have shown that individuals who were formerly type C children are at risk for substance abuse and eating disorders. For example, Miljkovitch et al. (2005) observed that drug misusers scored low in secure attachment cognitions and that both the lack of secure attachment and the preoccupied attachment cognitions of these individuals were significantly associated with depression scores. Zachrisson and Skårderud (2010) observed a greater prevalence of insecure attachment in the eating disordered population.

All of these adolescent adaptations related to anxiety should be considered with great attention. Indeed, Olivardia et al. (2004) showed that this anxiety can continue into adulthood and lead to the use of appearance-enhancing drugs. The reinforcement of muscle bulk is clearly a defense against the collapse of identity and a way of maintaining a body image experienced as a tonic mass that gives the feeling of existing (Gantheret, 1968). This anxiety can also emerge at different hard periods of life and not in adolescence. For example, Coquet (2016) identified “introspective conversion” to bodybuilding in individuals that had lived specific events in their lives and felt pushed to re-elaborate themselves. After some years of practice and the use of appearance-enhancing drugs, a new feeling of freedom, a sense of achievement, a positive response to the gaze of others, and the conviction of having control over their own lives emerged in these people.

## Intervention – Prevention

In this article, we show that the use of appearance-enhancing drugs is linked to an enacted body image that is the sense of making sporting activity brought forth in situation. We show that the aspect of appearance in body image is instrumental. Thus, appearance is not only driven by social stereotypes that can generate psychopathological disorders, but also by the sense of practices in which people are engaged that are recognized by peers, thus constituting ecological niches or *Umwelt* (e.g., von Uexküll et al., 1984; Froese and Di Paolo, 2011; Froese and Stewart, 2012). We also show that the enacted body image, which promotes embedded and embodied “being and acting in a world,” originates in the developmental history of each individual. The outcome of this development may re-emerge in specific and difficult periods of the life course. Thus, prevention and intervention efforts should be conceived in two directions: (a) promoting a safe instrumental use of the body in sports and (b) and helping those experiencing difficulties.

The promotion of safe instrumental body use in sports is a key way to prevent the negative effects of enacting a body image that is too excessive and specific (Hauw, 2017). This is related to building cultures of practice that maintain and value the positive experience of sports practice (e.g., Benson, 2003; Dworkin et al., 2003; Petitpas et al., 2005; Edwards et al., 2007). Recent models of talent development in sport such as, for example, the Developmental Model of Sport Participation (e.g., Côté and Fraser-Thomas, 2007) have particularly emphasized the importance of early sampling by participating in a wide range of sports and deliberate play. This allows children to experience different social interactions with peers and adults (i.e., coaches and parents) and reinforces the adaptation of emotional and self-regulating skills that can be positively invested in one sport in the future (Côté et al., 2009). In addition, longitudinal studies have found that youth who participated in various activities score more favorably on personal and social outcome measures such as well-being (Busseri et al., 2006) and positive peer relationships (Fredricks and Eccles, 2006). Hence, the construction of a well-suited enacted body image is scaffolded during youth development by various practices and specific forms of training that prevent weaknesses from emerging at later stages.

To help athletes experiencing difficulties, psychological services should be offered. The enactive approach suggests that the experience of being in the world is a historical and embedded process. Recent research has shown that this experience can be modified through specific interventions (e.g., Leahy and Harrigan, 2006; Creado and Reardon, 2014, 2016). The process of re-enactment consists of “reviving” a personal experience that links an activity to a situation using an artificial situation that reproduces the main elements at stake in this experience. For example, self-confrontation, elicitation, and life-course interviews have been used to put athletes in a situation to re-enact previous experience in a sports situation (e.g., Hauw and Durand, 2004; Hauw and Bilard, 2012; Villemain and Hauw, 2014; Hauw and Mohamed, 2015; Gesbert et al., 2017; Rochat et al., 2017). While these re-enactment methods have mainly been used for performance analysis, they can also be used to help individuals to modify this activity-situation interaction and generate a new way of experiencing or sense-making. Thus, it is in this direction that we suggest the two main forms of re-enactment be used to help athletes experiencing difficulties with substance use linked to body image: collective role games and individual life-course interviews. During collective role games, athletes are confronted with situations in which they have to show how they use these substances and explain why they do so to the other participants (from the same sport) and a sports psychologist (Bilard, 2017). The impact might be maximized by also including athletes who are not users. When sharing and comparing their experiences of acting in such re-enactment situations, they reveal their pre-reflective experience in contrast to those of others and, in doing so, they progressively gain awareness about what they do and gain access to other fields of possible conduct. This form of crossing experiences has been used in the workplace to resolve stress by changing the meaning of a situation and thus the practice (e.g., Duboscq and Clot, 2010). The life-course interview is another form of re-enactment that generates a gain of awareness. In the first part of the interview, it is important to re-build the history of past experience with the person by tracing marks in the story of development, its important events and successes and failures. In the second part of the interview, the athlete then re-lives the experience regarding these marks of personal history (see for a detailed methodology, Hauw and Bilard, 2012). In doing so, the athlete is able to re-enact his or her body experience at the pre-reflective level and with the help of distance from the past experience, and the sports psychologist is positioned to offer new senses of the interaction they experienced between their activity and situation. This method has been used for cases of doping and alcohol abuse in elite athletes and for career development (Hauw and Lemeur, 2013; Hauw, in review) and looks like narrative therapy that was also used in sport psychology (e.g., Denison and Winslade, 2006; Tamminen and Bennett, 2017). In addition to these methods, a free hotline and remote services are useful to identify those in need of help. Remote resources provide help at a distance via discussions, email and websites, with a notable example being Adam Winstock’s Global Drug Survey, which provides information on safe drug use with the High–Way Code (Winstock, 2016). Ecoute Dopage found that 23% of the callers reporting anabolic steroid use requested “help” in making the decision to stop using these drugs, to reduce use, to find alternatives, or to begin weaning off them (Bilard; 2000–2013; Palmié, 2010).

## Conclusion

In this paper, we have looked at appearance-enhancing drug use in sport related to body image. Through an enactive lens, we propose a dynamic, flexible and embedded account of body image that offers the possibility of reconnecting what emerges at the level of individual experience with the way sport and physical activities are performed through the sense-making processes that are brought forth. Theoretical conceptualizations, clinical observations, studies and illustrations were used to ground and draw this framework. Empirical evidence is now needed to support this framework in relation to the specific issue of appearance-enhancing drug use. Regarding the complexity and multi-facets of the enacted body image, future research will nevertheless also have to consider the preservation of the embodied, embedded and dynamic properties of human activities and experience in the methodology they employ to determine the types of sense-making in sport that foster the emergence of problematic appearance-enhancing substances drug use.

## Author Contributions

All authors listed have made a substantial, direct and intellectual contribution to the work, and approved it for publication.

## Conflict of Interest Statement

The authors declare that the research was conducted in the absence of any commercial or financial relationships that could be construed as a potential conflict of interest.
